# Supplementary material for: HIV-Induced Hyperactivity of Striatal Neurons Is Associated with Dysfunction of Voltage-Gated Calcium and Potassium Channels at Middle Age
Source: Membranes (Basel). 2022 Jul 28;12(8):737. doi: 10.3390/membranes12080737 (PMC9415409; doi:10.3390/membranes12080737)

Supplementary Information – for ID#: Membranes-1813204

Title: HIV-induced hyperactivity of striatal neurons is associated with dysfunction of voltage-gated calcium and potassium channels in rats at middle age

Authors: Christina E. Khodr*, Lihua Chen*, Lena Al-Harthi, Xiu-Ti Hu

*Authors have equal contribution to this study.

Supplementary Figure S1. The full molecular weight (MW) ranges of the membranes probed for $Ca_v1.2$ and $Ca_v1.3$ are shown for the representative samples shown in Figure 2D of the main text. All membranes were cut in half to facilitate multiple probing for proteins of interest. **A)** The full MW ranges of membranes probed for $Ca_v1.2$ (high MW portion) and actin (low MW portion) are shown. For the representative $Ca_v1.2$ bands, the samples were run on the same gel, but were inverted compared with the order shown; ie, the bands were rearranged to achieve the desired order for presentation. **B)** The full MW ranges of membranes probed for $Ca_v1.3$ (high MW portion) and actin (low MW portion) are shown.

Supplementary Figure S2. The full molecular weight (MW) ranges of the membranes probed for NMDAR2B and $GABA_AR\beta2,3$ are shown for the representative samples shown in Figure 3A of the main text. All membranes were cut in half to facilitate multiple probing for proteins of interest. **A)** The full MW ranges of membranes probed for NMDAR (high MW portion) and actin (low MW portion) are shown. For the representative NMDAR bands, the samples were run on the same gel, but were not side-by-side; ie, the bands shown were cropped from two different areas of the same membrane. **B)** The full MW ranges of membranes probed for $GABA_AR\beta2,3$ and actin (both low MW portions) are shown. The membrane was first probed for $GABA_AR\beta2,3$, then stripped and re-probed for actin.

Supplementary Figure S1.

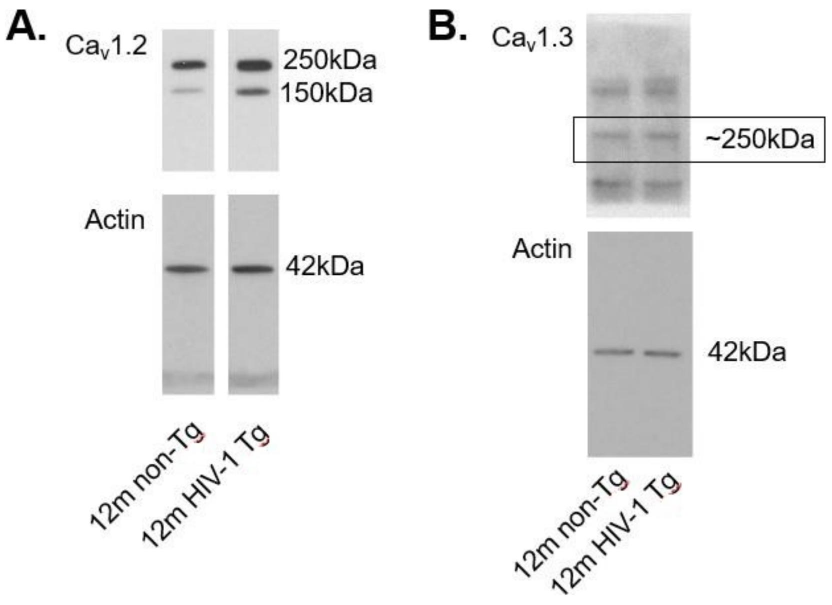

Supplementary Figure S2.

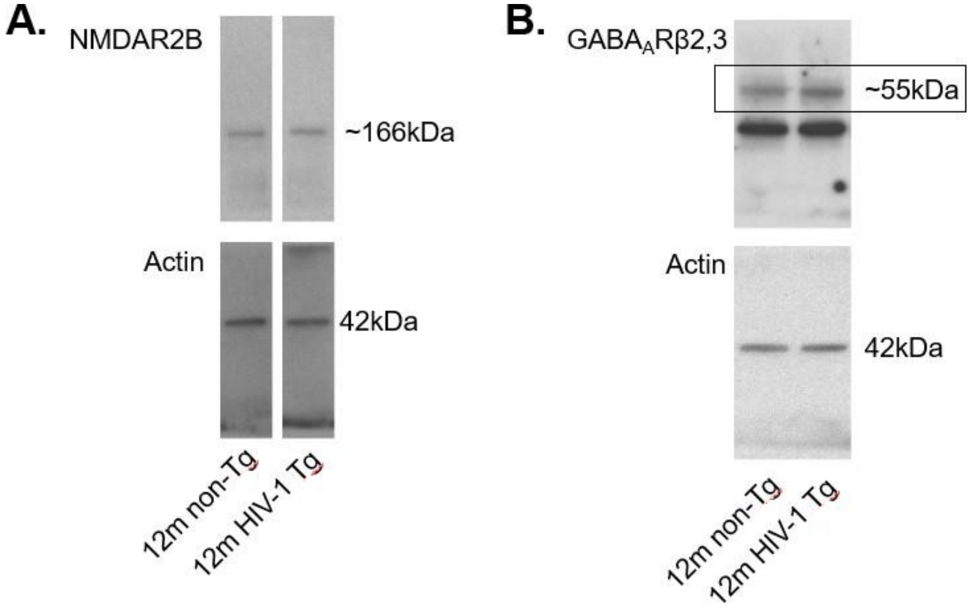

Supplement: Supplementary file 1 [file membranes-12-00737-s001.zip › membranes-1813204-supplementary.pdf]
